# 

**Published:** 1873-02-15

**Authors:** 


### Medical Examiner.

A Semi-Monthly Journal of Medical Sciences.

No. 4. CHICAGO, FEBRUARY 15, 1873. Vol. XIV.

TERMS.—Yearly Subscriptions, Three Dollars, in advance. Single Numbers Fifteen Cents.
All Communications should be addressed to Publishers Medical Examiner, 792 Wabash Av., Chicago.

